# Supplementary material for: A novel transcript of MEF2D promotes myoblast differentiation and its variations associated with growth traits in chicken
Source: PeerJ. 2020 Feb 4;8:e8351. doi: 10.7717/peerj.8351 (PMC7006513; doi:10.7717/peerj.8351)
Supplement: Supplemental Information 1 [file peerj-08-8351-s001.doc]

**Table S1.** **Information of MEF2D protein sequence in various organisms.**

| Accession number | Organism | Size（AA） |
| --- | --- | --- |
| NP_001026771.2 | *Gallus gallus* | 518 |
| NP_005911 | *Homo sapiens* | 521 |
| NP_001192107.1 | *Bos taurus* | 507 |
| NP_598426.1 | *Mus musculus* | 507 |
| NP_110487.2 | *Rattus norvegicus* | 507 |
| NP_571392.1 | *Danio rerio* | 529 |
| NP_001072927.1 | *Xenopus tropicalis* | 496 |
| XP_013852412.1 | *Sus scrofa* | 509 |
| XP_015739889.1 | *Coturnix japonica* | 518 |
| XP_010722165.1 | *Meleagris gallopavo* | 517 |

**Table S2.** **Detail information of primers for gene cloning, overexpression and qPCR.**

| **ID_primer name** | **Nucleotide sequences (5’→3’）** | **Use** |
| --- | --- | --- |
| MEF2D-C | F: GCTGAGCGTGTTGTGCGAC  R: TCGCCCGTTGTGCTAGAAT | Gene cloning |
| RBFOX2-O | F: GAATTCATGGCGGAGGGCGGCCAGCC  R:GGATCCTCACGTCACTTCAGTAGGGGGC | Overexpression |
| MEF2D-O | F:GAATTCATGGGGAGGAAAAAGATCCAG  R: GGATCCTTATGTGACCCAGGTATCCAAC | Overexpression |
| RBFXO2-Q | F: GACTTCAGGTTATCATGGA  R: GTAGAGCGTCAGATTGTGC | qPCR |
| MEF2D-Q1 | F: GAGCGGCAAAGGGCTGATG  R: GTGTTGTATGCGGTCGGCA | qPCR |
| MEF2D-Q2 | F: GCCAGCCAGTGCAGGGGCGAT  R: CCCAGAATCCCAAAGTGCTGCC | qPCR |
| MEF2D-Q3 | F: TTGATGGAGGATAAATACC  R: CAGGCTCGGGGTGGCAAAGT | qPCR |
| MEF2D-Q4 | F: CGGAGGATCACTTGGCTCTGA  R: TGTTGTATGCGGTCGGCAT | qPCR |
| MYOD | F: GCTACTACACGGAATCACCAAAT  R: CTGGGCTCCACTGTCACTCA |  |
| MYOG | F: CGGAGGCTGAAGAAGGTGAA  R: CGGTCCTCTGCCTGGTCAT | qPCR |
| MHC  (MYHB1) | F: CTCCTCACGCTTTGGTAA  R: TGATAGTCGTATGGGTTGGT | qPCR |
| GAPDH | F: CAGAACATCATCCCAGCGT  R: CAGGTCAGGTCAACAACAG | qPCR |
| 18SrRNA | F: TGCATGTCTAAGTACACACGG  R: AGGTCGGCGCTCGTCGGCATG | qPCR |

**Table S3.** **Detail information of primers for SNP identification.**

| **ID_primer name** | **Nucleotide sequences (5’→3’）** | **Annealing temperature (℃)** |
| --- | --- | --- |
| PM1 | F: CGCAGGAACTGGAGATGGGT  R: TGGGATTGGAAAATGGGGTG | 60 |
| PM2 | F: CTCCTGCCGTTACCTTCCTT  R: ATTCCCCTCAGATCCTTCCC | 60 |
| PM3 | F: CCCTCCATCTGTCTGTCTGTCC  R: CACTCCCTGTGCTTCTTGTTCA | 63 |
| PM4 | F: CCTCCATCTACCCGCTCCCCT  R: ACTCAGCCCCTCGCATTGCCT | 63 |
| PM5 | F: TGGAACAAAATAGCCACAG  R: CCACAAAACACACCACCTC | 58 |
| PM6 | F: CAGGGGTCGTGCTGAAGGC  R: GCAAAGGGAAACGCAAAGG | 63 |
| PM7 | F: TAGCTGGTTTTGTTTTAGC  R: CTTTGTTTTTCATCTCGTG | 59 |
| PM8 | F: TGTATGTCCGTGGTTGTGT  R: CTCTTCCTCTGCTGTGTGA | 58 |
| PM9 | F: CCACGCTCTGATGGTTTTGTA  R: CTCCGTCTCCCTTTGATTTCT | 62 |
