# Supplementary material for: A novel transcript of MEF2D promotes myoblast differentiation and its variations associated with growth traits in chicken
Source: PeerJ. 2020 Feb 4;8:e8351. doi: 10.7717/peerj.8351 (PMC7006513; doi:10.7717/peerj.8351)
Supplement: Supplemental Information 2 [file peerj-08-8351-s002.docx]

**MEF2D-V1**

The transcript V1 was 3222 bp in length, a deletion of 889 bp (1446-2334 nt), and predicted to encode 251 AA.

**cDNA sequence (**3222 nt**)**

gtgtgccctggggcctcaggggacctgcagctcccgctgccccccgtgccctcccggcttttcccttcccactctcgggatgaagagcccgagaggtaacaaactcatcgggcgtaatgaagtggtggctcgaggcagggacgcacctgaggagcctctgtgttttgtgggtggatggtttggggccggctctgttagccgtgatgagggatggtgaagagggttcagcccagggggaagtttgggtttggaacccaaatgttttgtggccccggacccgttgctagaggaaacgtggaaaggggaaggagggattgtggttgggcagctcttcccctctgcaatctgagcctccgtcttggcccctccacctccccttggcacggaccgtgccccccgagcgctcggtgtccccacgtcgccatgggctggcggtggccctggggtcacattgccacgcgcggagctccctgcagccgggctttcccatggcggtgctacggaggtttctgatggctcagccccactggattcgccggagggtgccatgaactgagcgcccagctccgtttcgtttgctttcctttggttcttcatcgctccctctcccgtgcatttttcatgagaacgttttaaaagggggttcctctcgcactccccccccccccccccccgagcgacgctgtgctgctccggtgaggatctcctgagaagATGGGGAGGAAAAAGATCCAGATCCAGCGGATCACGGATGAGCGGAACCGGCAGGTGACCTTCACCAAGCGTAAGTTCGGCTTGATGAAGAAAGCCTACGAGCTGAGCGTGTTGTGCGACTGCGAGATCGCCCTGATCATTTTCAACCACTCCAACAAGCTGTTCCAGTATGCCAGCACCGACATGGACAAGGTGCTGCTTAAGTACACTGAGTACAACGAGCCCCATGAGAGCCGGACCAATGCGGACATCATTGAGACGTTAAGAAAGAAAGGTTTTAATGGCTGTGACAGCCCGGAACCCGACGGAGATGACTCGATAGACCAGAGCCCCTTGATGGAGGATAAATACCGTAAAGGCAGCGAGGATCTGGATATCCTGTTCAAGCGATACGGTTCCGCAGTGCCCACGCCAAACTTTGCCATGCCAGTGACGGTTCCCGTGACCAACCAGAACACACTGCAGTTCAGCAGCCCGGGCAGCTCGTTGGTGACCCAATCCTTGGTGACCTCGTCGCTGACCGACCCCCGGCTCCTCTCCCCACAGCAGCCAACACTGCAGAGGAATACGGTGTCCCCAGGGCTGCCGCAGCGGCCAGCCAGTGCAGGGGCGATGCTTGGGGGTGACCTGAACAACACCAACGGAGCCTGCCCGAGCCCCGTGGGCAACGGCTACGTGAGTGCTCGTGCCTCCCCCGGTCTCCTTCCCGTCTCCAACGGCAGCACTTTGGGATTCTGGGGACAAAACGGGTTTTAGgggttggggtggggagattatcataaactgcctgagaaatagctttaggattttgtaggcattcattctagcacaacgggcgacgcacgcagttcgtacagctgggagctgcaagttccagggtgggtggcacagaaatcaaagggagacggagcggagatgggggccgtggggctgcgctggtgcagcgatgcgggcactgagcctccccgatggctgccccacagcctgggcaggggaggatggttcagccccacggaaagaggctgcgtgctgcagcatcgcttgga

ggcggggggggggaacacgagacaggtttaaagcttgatctttgctgtttgggtgagaggtggttgtggacgtcgtccttccctgcagagaagggagcacgtctgcttctggccacggtcatccaaaggctgcagccacctctgctgaggtcccactgagccccgcagggtgctcggctgtggcagggctggtttgggagaaacagtggttttaccaaacaaaacgtgcaggtgtgtttaaaacgcgttccctgctctatatgtgtgtcaaagctttaagtcagaagctaggagtaaatcgttgttatttaagggttgccactgaaataacacccttagggattttgcctgaaggcctcggaaggagttgagctgagctgtgcaggatctctgcagcagcctcagccacttggttcagtggaaggaaaaagggcacgagggaagcaggggaagttctggagctgcttcgcggtgccatctccgcccccatccctgctcaggatagtgaggacagtcctcacaccacaccattcctgtccactgaagcttgtggttgcagaatggcacagaacctgccccaacacctgaactcatcctgctccagggccacagggaggtccctgggggatgggggctgcgcgtctgcctccttccagagaccccagcagagttagaggggttccagcccaacctgttgcagcgggacagattgtaatggaatgaatgtgggcaacttaaggcgagcgaataggggaagtcctccgaaaatccgggtgggagtgaatggtgatggctgtggagaggcagcacggagccgacgcagccccacatccctgcgcagcgctgcagaggcgtgcaggcagcaatctcctgctgaaaacctttgagaagccccatttgccaccgtttatccataggccgattgagttctgaatgtgaaatgttggcgttcctctcccattgcggcacgcagtgggggacccacgcgggcagattctgtgccgtgtaccccaatcctcctctctccgcctcaaacccttcacggtgtgcaccgggcttcttgtccacgccgaggtgacgtggatctgtccccacgtcccaactccgtccctctgtcccagctctgtccccgtgtccatccaggaagcagccacaccgccagcacccggcggccctgcgtgcatcggacccgcacggcagctctttgcacggcctcgctttggaaattaggtgggattgacgatgtctcgtcgctctcatttccttttctttttttcctgaccgtcctgcaagaaaaaataacgccgcaaccgaacgttttccggcccctcaaaggagcgggagggcggcagtgcggcgcttggagggcgacggaacatttctgctcattgacttcacgccctgacttcacatctcagagagccagaaaaaaaaaaaggaaaaaaaatccaaaaaaaaaaaa

Note: The capital letters are coding DNA sequence; the letters with underline are primers for gene cloning.

**Protein sequence (**251 aa**)**

MGRKKIQIQRITDERNRQVTFTKRKFGLMKKAYELSVLCDCEIALIIFNHSNKLFQYASTDMDKVLLKYTEYNEPHESRTNADIIETLRKKGFNGCDSPEPDGDDSIDQSPLMEDKYRKGSEDLDILFKRYGSAVPTPNFAMPVTVPVTNQNTLQFSSPGSSLVTQSLVTSSLTDPRLLSPQQPTLQRNTVSPGLPQRPASAGAMLGGDLNNTNGACPSPVGNGYVSARASPGLLPVSNGSTLGFWGQNGF

Note: MADS-Box (2-57 aa) and MEF2-domain (58-86 aa).

**MEF2D-V2**

The transcript V2 was 3616 bp in length, a deletion of 498 bp (1139-1636 nt) and predicted to encode 353 AA..

**cDNA sequence (**3616 nt**)**

gtgtgccctggggcctcaggggacctgcagctcccgctgccccccgtgccctcccggcttttcccttcccactctcgggatgaagagcccgagaggtaacaaactcatcgggcgtaatgaagtggtggctcgaggcagggacgcacctgaggagcctctgtgttttgtgggtggatggtttggggccggctctgttagccgtgatgagggatggtgaagagggttcagcccagggggaagtttgggtttggaacccaaatgttttgtggccccggacccgttgctagaggaaacgtggaaaggggaaggagggattgtggttgggcagctcttcccctctgcaatctgagcctccgtcttggcccctccacctccccttggcacggaccgtgccccccgagcgctcggtgtccccacgtcgccatgggctggcggtggccctggggtcacattgccacgcgcggagctccctgcagccgggctttcccatggcggtgctacggaggtttctgatggctcagccccactggattcgccggagggtgccatgaactgagcgcccagctccgtttcgtttgctttcctttggttcttcatcgctccctctcccgtgcatttttcatgagaacgttttaaaagggggttcctctcgcactccccccccccccccccccgagcgacgctgtgctgctccggtgaggatctcctgagaagATGGGGAGGAAAAAGATCCAGATCCAGCGGATCACGGATGAGCGGAACCGGCAGGTGACCTTCACCAAGCGTAAGTTCGGCTTGATGAAGAAAGCCTACGAGCTGAGCGTGTTGTGCGACTGCGAGATCGCCCTGATCATTTTCAACCACTCCAACAAGCCGTTCCAGTATGCCAGCACCGACATGGACAAGGTGCTGCTTAAGTACACTGAGTACAACGAGCCCCATGAGAGCCGGACCAATGCGGACATCATTGAGACGTTAAGAAAGAAAGGTTTTAATGGCTGTGACAGCCCGGAACCCGACGGAGATGACTCGATAGACCAGAGCCCCTTGATGGAGGATAAATACCGTAAAGGCAGCGAGGATCTGGATATCCTGTTCAAGCGATACGGTTCCGCAGTGCCCACGCCAAACTTTGCCACCCCGAGCCTGCTGACACAGGGGCTGCCGTTCTCCGCCATGCCGACCGCATACAACACAGATTATCAGCTGACGAGTGCTGAGTTATCTTCGCTGCCAGCATTCAGCTCACCTGGTGGGTTGTCCCTTGGCAACATCTCTGCCTGGCAGCAGCAGCAGCAGCAGCAGCAGCAGCAGCGACAGCAGCAGCAGCAGCAGCAACAACAGCAACAGCAGCAGCAGCAGCAACAACAGCAGCAGCAGCAGCAGCAACAGCAGCAGCAACAACAGCAGCAGCAGCACCTGGTTCCTGTATCACTAGGAAATTTAATACAAGGGAGCCACTTGTCCCACACCACCACTTTGACTGTCAACACCAACCCCAACATCAGCATCAAGTCGGAGCCCGTCTCGCCCAACCGGGAACGAAACACTGCCACCCCACTCAGCACCTTCCCCCACCAGCCCCGCCATGAGCCCACCGGCCGCTCGCCTGTCGACAGCCTCAGCAGCAACACCAGCTCCTACGAGGGCAGCGAGCGAGACGATCCCACTCGCACCGACTTCAGCTCCACCCTGGGGCTGCTGCACCCTGGTGGTGAGCCCGAGGGGGAGAGCCCTTCGGTGAAACGCATGCGGTTGGATACCTGGGTCACATAAcgggtccctatcatccccgggagcgccgctggcttgcagcggggaggggaggggggggttgggattctggggacaaaacgggttttaggggttggggtggggagattatcataaactgcctgagaaatagctttaggattttgtaggcattcattctagcacaacgggcgacgcacgcagttcgtacagctgggagctgcaagttccagggtgggtggcacagaaatcaaagggagacggagcggagatgggggccgtggggctgcgctggtgcagcgatgcgggcactgagcctccccgatggctgccccacagcctgggcaggggaggatggttcagccccacggaaagaggctgcgtgctgcagcatcgcttggaggcggggggggggaacacgagacaggtttaaagcttgatctttgctgtttgggtgagaggtggttgtggacgtcgtccttccctgcagagaagggagcacgtctgcttctggccacggtcatccaaaggctgcagccacctctgctgaggtcccactgagccccgcagggtgctcggctgtggcagggctggtttgggagaaacagtggttttaccaaacaaaacgtgcaggtgtgtttaaaacgcgttccctgctctatatgtgtgtcaaagctttaagtcagaagctaggagtaaatcgttgttatttaagggttgccactgaaataacacccttagggattttgcctgaaggcctcggaaggagttgagctgagctgtgcaggatctctgcagcagcctcagccacttggttcagtggaaggaaaaagggcacgagggaagcaggggaagttctggagctgcttcgcggtgccatctccgcccccatccctgctcaggatagtgaggacagtcctcacaccacaccattcctgtccactgaagcttgtggttgcagaatggcacagaacctgccccaacacctgaactcatcctgctccagggccacagggaggtccctgggggatgggggctgcgcgtctgcctccttccagagaccccagcagagttagaggggttccagcccaacctgttgcagcgggacagattgtaatggaatgaatgtgggcaacttaaggcgagcgaataggggaagtcctccgaaaatccgggtgggagtgaatggtgatggctgtggagaggcagcacggagccgacgcagccccacatccctgcgcagcgctgcagaggcgtgcaggcagcaatctcctgctgaaaacctttgagaagccccatttgccaccgtttatccataggccgattgagttctgaatgtgaaatgttggcgttcctctcccattgcggcacgcagtgggggacccacgcgggcagattctgtgccgtgtaccccaatcctcctctctccgcctcaaacccttcacggtgtgcaccgggcttcttgtccacgccgaggtgacgtggatctgtccccacgtcccaactccgtccctctgtcccagctctgtccccgtgtccatccaggaagcagccacaccgccagcacccggcggccctgcgtgcatcggacccgcacggcagctctttgcacggcctcgctttggaaattaggtgggattgacgatgtctcgtcgctctcatttccttttctttttttcctgaccgtcctgcaagaaaaaataacgccgcaaccgaacgttttccggcccctcaaaggagcgggagggcggcagtgcggcgcttggagggcgacggaacatttctgctcattgacttcacgccctgacttcacatctcagagagccagaaaaaaaaaaaggaaaaaaaatccaaaa

aaaaaaaa

Note: The capital letters are coding DNA sequence; the letters with underline are primers for gene cloning.

**Protein sequence (**353 aa**)**

MGRKKIQIQRITDERNRQVTFTKRKFGLMKKAYELSVLCDCEIALIIFNHSNKLFQYASTDMDKVLLKYTEYNEPHESRTNADIIETLRKKGFNGCDSPEPDGDDSIDQSPLMEDKYRKGSEDLDILFKRYGSAVPTPNFATPSLLTQGLPFSAMPTAYNTDYQLTSAELSSLPAFSSPGGLSLGNISAWQQQQQQQQQQRQQQQQQQQQQQQQQQQQQQQQQQQQQQQQQQQQHLVPVS

LGNLIQGSHLSHTTTLTVNTNPNISIKSEPVSPNRERNTATPLSTFPHQPRHEPTGRSPVDSL

SSNTSSYEGSERDDPTRTDFSSTLGLLHPGGEPEGESPSVKRMRLDTWVT

Note: MADS-Box (2-57 aa) and MEF2-domain (58-86 aa).

**MEF2D-V3**

The transcript V3 was 4135 bp in length, inserting a 21 bp after exon 8 (1570 nt) and AAC insertion at 1813 nt, predicting encode 526 AA.

**cDNA sequence (**4135 nt**)**

gtgtgccctggggcctcaggggacctgcagctcccgctgccccccgtgccctcccggcttttcccttcccactctcgggatgaagagcccgagaggtaacaaactcatcgggcgtaatgaagtggtggctcgaggcagggacgcacctgaggagcctctgtgttttgtgggtggatggtttggggccggctctgttagccgtgatgagggatggtgaagagggttcagcccagggggaagtttgggtttggaacccaaatgttttgtggccccggacccgttgctagaggaaacgtggaaaggggaaggagggattgtggttgggcagctcttcccctctgcaatctgagcctccgtcttggcccctccacctccccttggcacggaccgtgccccccgagcgctcggtgtccccacgtcgccatgggctggcggtggccctggggtcacattgccacgcgcggagctccctgcagccgggctttcccatggcggtgctacggaggtttctgatggctcagccccactggattcgccggagggtgccatgaactgagcgcccagctccgtttcgtttgctttcctttggttcttcatcgctccctctcccgtgcatttttcatgagaacgttttaaaagggggttcctctcgcactccccccccccccccccccgagcgacgctgtgctgctccggtgaggatctcctgagaagATGGGGAGGAAAAAGATCCAGATCCAGCGGATCACGGATGAGCGGAACCGGCAGGTGACCTTCACCAAGCGTAAGTTCGGCTTGATGAAGAAAGCCTACGAGCTGAGCGTGTTGTGCGACTGCGAGATCGCCCTGATCATTTTCAACCACTCCAACAAGCTGTTCCAGTATGCCAGCACCGACATGGACAAGGTGCTGCCTAAGTACACTGAGTACAACGAGCCCCATGAGAGCCGGACCAATGCGGACATCATTGAGACGTTAAGAAAGAAAGGTTTTAATGGCTGTGACAGCCCGGAACCCGACGGAGATGACTCGATAGACCAGAGCCCCTTGATGGAGGATAAATACCGTAAAGGCAGCGAGGATCTGGATATCCTGTTCAAGCGATACGGTTCCGCAGTGCCCACGCCAAACTTTGCCATGCCAGTGACGGTTCCCGTGACCAACCAGAACACACTGCAGTTCAGCAGCCCGGGCAGCTCGTTGGTGACCCAATCCTTGGTGACCTCGTCGCTGACCGACCCCCGGCTCCTCTCCCCACAGCAGCCAACACTGCAGAGGAATACGGTGTCCCCAGGGCTGCCGCAGCGGCCAGCCAGTGCAGGGGCAATGCTTGGGGGTGACCTGAACAACACCAACGGAGCCTGCCCGAGCCCCGTGGGCAACGGCTACGTGAGTGCTCGTGCCTCCCCCGGACTCCTTCCCGTCTCCAACGGCAGCACTTTGGGCAAGATCATCCCGGCCAAATCCCCTCCGCCGCCACCCCACAGCGCCCAGCTCGCCTCCAACAGCCGCAAGCCGGATCTGCGCGTCATCACCTCGCAGAGCGGCAAAGGGCTGATGCACCACCTGACGGAGGATCACTTGGCTCTGAACACGCAGCGGTTAGGTGTCTCCCAAGCGACTCACTCTCTAACCACGCCGGTTGTTTCTGTTGCCACCCCGAGCCTGCTGACACAGGGGCTGCCGTTCTCCGCCATGCCGACCGCATACAACACAGATTATCAGCTGACGAGTGCTGAGTTATCTTCGCTGCCAGCATTCAGCTCACCTGGTGGGTTGTCCCTTGGCAACATCTCTGCCTGGCAGCAGCAGCAGCAGCAGCAGCAGCAGCAGCAACAGCAGCAGCAGCAGCAGCAACAACAGCAACAGCAGCAGCAGCAGCAACAACAGCAGCAGCAGCAGCAGCAACAGCAGCAGCAACAACAGCAGCAGCAGCACCTGGTTCCTGTATCACTAGGAAATTTAATACAAGGGAGCCACTTGTCCCACACCACCACTTTGACTGTCAACACCAACCCCAACATCAGCATCAAGTCGGAGCCCGTCTCGCCCAACCGGGAACGAAACACTGCCACCCCACTCAGCACCTTCCCCCACCAGCCCCGCCATGAGCCCACCGGCCGCTCGCCTGTCGACAGCCTCAGCAGCAACACCAGCTCCTACGAGGGCAGCGAGCGAGACGATCCCACTCGCACCGACTTCAGCTCCACCCTGGGGCTGCTGCGCCCTGGTGGTGAGCCCGAGGGGGAGAGCCCTTCGGTGAAACGCATGCGGTTGGATACCTGGGTCACATAAcgggtccctatcatccccgggagcgccgctggcttgcagcggggaggggaggggggggttgggattctggggacaaaacgggttttaggggttggggtggggagattatcataaactgcctgagaaatagctttaggattttgtaggcattcattctagcacaacgggcgacgcacgcagttcgtacagctgggagctgcaagttccagggtgggtggcacagaaatcaaagggagacggagcggagatgggggccgtggggctgcgctggtgcagcgatgcgggcactgagcctccccgatggctgccccacagcctgggcaggggaggatggttcagccccacggaaagaggctgcgtgctgcagcatcgcttggaggcggggggggggaacacgagacaggtttaaagcttgatctttgctgtttgggtgagaggtggttgtggacgtcgtccttccctgcagagaagggagcacgtctgcttctggccacggtcatccaaaggctgcagccacctctgctgaggtcccactgagccccgcagggtgctcggctgtggcagggctggtttgggagaaacagtggttttaccaaacaaaacgtgcaggtgtgtttaaaacgcgttccctgctctatatgtgtgtcaaagctttaagtcagaagctaggagtaaatcgttgttatttaagggttgccactgaaataacacccttagggattttgcctgaaggcctcggaaggagttgagctgagctgtgcaggatctctgcagcagcctcagccacttggttcagtggaaggaaaaagggcacgagggaagcaggggaagttctggagctgcttcgcggtgccatctccgcccccatccctgctcaggatagtgaggacagtcctcacaccaca

ccattcctgtccactgaagcttgtggttgcagaatggcacagaacctgccccaacacctgaactcatcctgctccagggccacagggaggtccctgggggatgggggctgcgcgtctgcctccttccagagaccccagcagagttagaggggttccagcccaacctgttgcagcgggacagattgtaatggaatgaatgtgggcaacttaaggcgagcgaataggggaagtcctccgaaaatccgggtgggagtgaatggtgatggctgtggagaggcagcacggagccgacgcagccccacatccctgcgcagcgctgcagaggcgtgcaggcagcaatctcctgctgaaaacctttgagaagccccatttgccaccgtttatccataggccgattgagttctgaatgtgaaatgttggcgttcctctcccattgcggcacgcagtgggggacccacgcgggcagattctgtgccgtgtaccccaatcctcctctctccgcctcaaacccttcacggtgtgcaccgggcttcttgtccacgccgaggtgacgtggatctgtccccacgtcccaactccgtccctctgtcccagctctgtccccgtgtccatccaggaagcagccacaccgccagcacccggcggccctgcgtgcatcggacccgcacggcagctctttgcacggcctcgctttggaaattaggtgggattgacgatgtctcgtcgctctcatttccttttctttttttcctgaccgtcctgcaagaaaaaataacgccgcaaccgaacgttttccggcccctcaaaggagcgggagggcggcagtgcggcgcttggagggcgacggaacatttctgctcattgacttcacgccctgacttcacatctcagagagccagaaaaaaaaaaaggaaaaaaaatccaaa

aaaaaaaaa

Note: The capital letters are coding DNA sequence; the letters with underline are primers for gene cloning.

**Protein sequence (**526 aa**)**

MGRKKIQIQRITDERNRQVTFTKRKFGLMKKAYELSVLCDCEIALIIFNHSNKLFQYASTDMDKVLLKYTEYNEPHESRTNADIIETLRKKGFNGCDSPEPDGDDSIDQSPLMEDKYRKGS

EDLDILFKRYGSAVPTPNFAMPVTVPVTNQNTLQFSSPGSSLVTQSLVTSSLTDPRLLSPQQPTLQRNTVSPGLPQRPASAGAMLGGDLNNTNGACPSPVGNGYVSARASPGLLPVSNGSTLGKIIPAKSPPPPPHSAQLASNSRKPDLRVITSQSGKGLMHHLTEDHLALNTQRLGVSQATHSLTTPVVSVATPSLLTQGLPFSAMPTAYNTDYQLTSAELSSLPAFSSPGGLSLGNISAWQQQQQQQQQQQQQQQQQQQQQQQQQQQQQQQQQQQQQQQQQQQQHLVPVSLGNLIQGSHLSHTTTLTVNTNPNISIKSEPVSPNRERNTATPLSTFPHQPRHEPTGRSPVDSLSSNTSSYEGSE

RDDPTRTDFSSTLGLLRPGGEPEGESPSVKRMRLDTWVT

Note: MADS-Box (2-57 aa) and MEF2-domain (58-86 aa).

**MEF2D-V4**

The transcript V4 was 4132 bp in length, a 21 bp is inserted after exon 8 (1570 nt), and predicted to encode 526 AA. The position and sequence of exon 4 (973-1108 nt) of the transcript V4 is different from that of other transcripts.

**cDNA sequence (**4132 nt**)**

gtgtgccctggggcctcaggggacctgcagctcccgctgccccccgtgccctcccggcttttcccttcccactctcgggatgaagagcccgagaggtaacaaactcatcgggcgtaatgaagtggtggctcgaggcagggacgcacctgaggagcctctgtgttttgtgggtggatggtttggggccggctctgttagccgtgatgagggatggtgaagagggttcagcccagggggaagtttgggtttggaacccaaatgttttgtggccccggacccgttgctagaggaaacgtggaaaggggaaggagggattgtggttgggcagctcttcccctctgcaatctgagcctccgtcttggcccctccacctccccttggcacggaccgtgccccccgagcgctcggtgtccccacgtcgccatgggctggcggtggccctggggtcacattgccacgcgcggagctccctgcagccgggctttcccatggcggtgctacggaggtttctgatggctcagccccactggattcgccggagggtgccatgaactgagcgcccagctccgtttcgtttgctttcctttggttcttcatcgctccctctcccgtgcatttttcatgagaacgttttaaaagggggttcctctcgcactccccccccccccccccccgagcgacgctgtgctgctccggtgaggatctcctgagaagATGGGGAGGAAAAAGATCCAGATCCAGCGGATCACGGATGAGCGGAACCGGCAGGTGACCTTCACCAAGCGTAAGTTCGGCTTGATGAAGAAAGCCTACGAGCTGAGCGTGTTGTGCGACTGCGAGATCGCCCTGATCATTTTCAACCACTCCAACAAACTGTTCCAGTATGCCAGCACCGACATGGACAAGGTGCTGCTTAAGTACACTGAGTACAACGAGCCCCATGAGAGCCGGACCAATGCGGACATCATTGAGGCGCTGAACAAGAAGCACAGGGAGTGCGAAAGCCCGGAAGGGGATGAGTGTTTGCACTGACCCCGCAGACGGAGGAGAAATATAAAAAGATTGATGAGGAGTTTGATAAAATGATGCAGAGTTACCGGCTCGCATCCGCAGTGCCCACGCCAAACTTTGCCATGCCAGTGACGGCTCCCGTGACCAACCAGAACACACTGCAGTTCAGCAGCCCGGGCAGTTCGTTGGTGACCCAATCCTTGGTGACCTCGTCGCTGACCGACCCCCGGCTCCTCTCCCCACAGCAGCCAACACTGCAGAGGAATACGGTGTCCCCAGGGCTGCCGCAGCGGCCAGCCAGTGCAGGGGCGATGCTTGGGGGTGACCTGAACAACACCAACGGAGCCTGCCCGAGCCCCGTGGGCAACGGCTACGTGAGTGCTCGTGCCTCCCCCGGTCTCCTTCCCGTCTCCAACGGCAGCACTTTGGGCAAGATCATCCCGGCCAAATCCCTTCCGCCGCCACCCCACAGCACCCAGCTCGCCTCCAACAGCCGCAAGCCGGACCTGCGCGTCATCACCTCGCAGAGCGGCAAAGGGCTGATGCACCACCTGACGGAGGATCACTTGGCTCTGAACACGCAGCGGTTAGGTGTCTCCCAAGCGACTCACTCTCTAACCACGCCGGTTGTTTCTGTTGCCACCCCGAGCCTGCTGACACAGGGGCTGCCGTTCTCCGCCATGCCGACCGCATACAACACAGATTATCAGCTGACGAGTGCTGAGTTATCTTCGCTGCCAGCATTCAGCTCACCTGGTGGGTTGTCCCTTGGCAACATCTCTGCCTGGCAGCAGCAGCAGCAGCAGCAGCAGCAGCAGCAACAGCAGCAGCAGCAGCAGCAACAACAGCAACAGCAGCAGCAGCAGCAACAACAGCAGCAGCAGCAGCAGCAACAGCAGCAGCAACAACAGCAGTAGCAGCACCTGGTTCCTGTATCACTAGGAAATTTAATACAAGGGAGCCACTTGTCCCACACCACCACTTTGACTGTCAACACCAACCCCAACATCAGCATCAAGTCGGAGCCCGTCTCGCCCAACCGGGAACGAAACACTGCCACCCCACTCAGCACCTTCCCCCACCAGCCCCGCCATGAGCCCACCGGCCGCTCGCCTGTCGACAGCCTCAGCAGCAACACCAGCTCCTACGAGGGCAGCGAGCGAGACGATCCCACTCGCACCGACTTCAGCTCCACCCTGGGGCTGCTGCGCCCTGGTGGTGAGCCCGAGGGGGAGAGCCCTTCGGTGAAACGCATGCGGTTGGATACCTGGGTCACATAAcgggtccctatcatccccgggagcgccgctggcttgcagcggggaggggaggggggggttgggattctggggacaaaacgggttttaggggttggggtggggagattatcataaactgcctgagaaatagctttaggattttgtaggcattcattctagcacaacgggcgacgcacgcagttcgtacagctgggagctgcaagttccagggtgggtggcacagaaatcaaagggagacggagcggagatgggggccgtggggctgcgctggtgcagcgatgcgggcactgagcctccccgatggctgccccacagcctgggcaggggaggatggttcagccccacggaaagaggctgcgtgctgcagcatcgcttggaggcggggggggggaacacgagacaggtttaaagcttgatctttgctgtttgggtgagaggtggttgtggacgtcgtccttccctgcagagaagggagcacgtctgcttctggccacggtcatccaaaggctgcagccacctctgctgaggtcccactgagccccgcagggtgctcggctgtggcagggctggtttgggagaaacagtggttttaccaaacaaaacgtgcaggtgtgtttaaaacgcgttccctgctctatatgtgtgtcaaagctttaagtcagaagctaggagtaaatcgttgttatttaagggttgccactgaaataacacccttagggattttgcctgaaggcctcggaaggagttgagctgagctgtgcaggatctctgcagcagcctcagccacttggttcagtggaaggaaaaagggcacgagggaagcaggggaagttctggagctgcttcgcggtgccatctccgcccccatccctgctcaggatagtgaggacagtcctcacaccacaccattcctgtccactgaagcttgtggttgcagaatggcacagaacctgccccaacacctgaactcatcctgctccagggccacagggaggtccctgggggatgggggctgcgcgtctgcctccttccagagaccccagcagagttagaggggttccagcccaacctgttgcagcgggacagattgtaatggaatgaatgtgggcaacttaaggcgagcgaataggggaagtcctccgaaaatccgggtgggagtgaatggtgatggctgtggagaggcagcacggagccgacgcagccccacatccctgcgcagcgctgcagaggcgtgcaggcagcaatctcctgctgaaaacctttgagaagccccatttgccaccgtttatccataggccgattgagttctgaatgtgaaatgttggcgttcctctcccattgcggcacgcagtgggggacccacgcgggcagattctgtgccgtgtaccccaatcctcctctctccgcctcaaacccttcacggtgtgcaccgggcttcttgtccacgccgaggtgacgtggatctgtccccacgtcccaactccgtccctctgtcccagctctgtccccgtgtccatccaggaagcagccacaccgccagcacccggcggccctgcgtgcatcggacccgcacggcagctctttgcacggcctcgctttggaaattaggtgggattgacgatgtctcgtcgctctcatttccttttctttttttcctgaccgtcctgcaagaaaaaataacgccgcaaccgaacgttttccggcccctcaaaggagcgggagggcggcagtgcggcgcttggagggcgacggaacatttctgctcattgacttcacgccctgacttcacatctcagagagccagaaaaaaaaaaaggaaaaaaaatccaaaaaa

aaaaaa

Note: The capital letters are coding DNA sequence; the letters with underline are primers for gene cloning.

**Protein sequence (**526 aa**)**

MGRKKIQIQRITDERNRQVTFTKRKFGLMKKAYELSVLCDCEIALIIFNHSNKLFQYASTDMDKVLLKYTEYNEPHESRTNADIIEALNKKHRECESPEGDEVFALTPQTEEKYKKIDEEFDKMMQSYRLASAVPTPNFAMPVTAPVTNQNTLQFSSPGSSLVTQSLVTSSLTDPRLLSPQQPTLQRNTVSPGLPQRPASAGAMLGGDLNNTNGACPSPVGNGYVSARASPGLLPVSNGSTLGKIIPAKSLPPPPHSTQLASNSRKPDLRVITSQSGKGLMHHLTEDHLALNTQRLGVSQATHSLTTPVVSVATPSLLTQGLPFSAMPTAYNTDYQLTSAELSSLPAFSSPGGLSLGNISAWQQQQQQQQQQQQQQQQQQQQQQQQQQQQQQQQQQQQQQQQQQQHLVPVSLGNLIQGSHLSHTTTLTVNTNPNISIKSEPVSPNRERNTATPLSTFPHQPRHEPTGRSPVDSLSSNTSSYEGSE

RDDPTRTDFSSTLGLLRPGGEPEGESPSVKRMRLDTWVT

Note: MADS-Box (2-57 aa) and MEF2-domain (58-86 aa).
