# Supplementary figures and images for: A novel transcript of MEF2D promotes myoblast differentiation and its variations associated with growth traits in chicken
Source: PeerJ. 2020 Feb 4;8:e8351. doi: 10.7717/peerj.8351 (PMC7006513; doi:10.7717/peerj.8351)

**MEF2D**

**chr25:2,742,900-2,782,225**

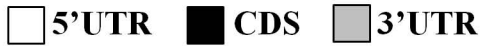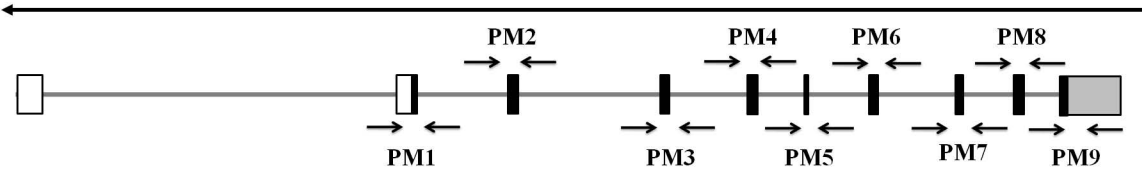

Supplement: Supplemental Information 3 [file peerj-08-8351-s003.pdf]

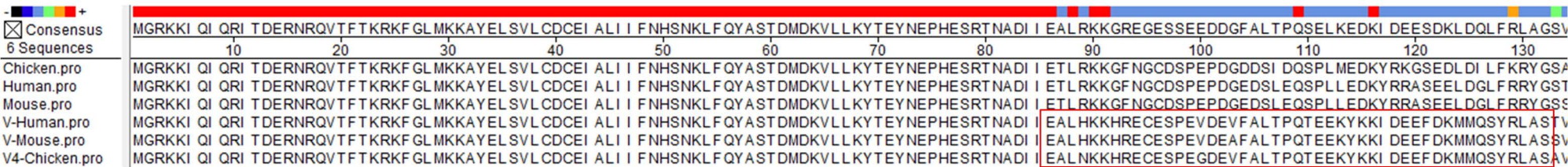

87-132 AA

Supplement: Supplemental Information 4 [file peerj-08-8351-s004.pdf]

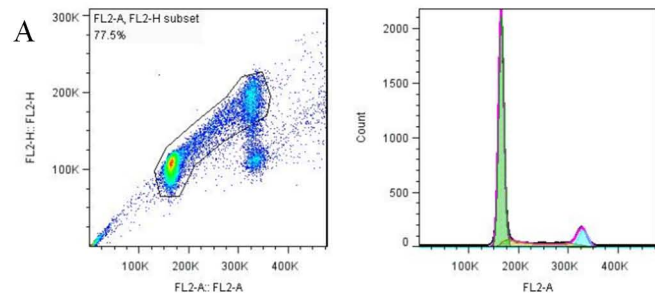

EGFP-Control

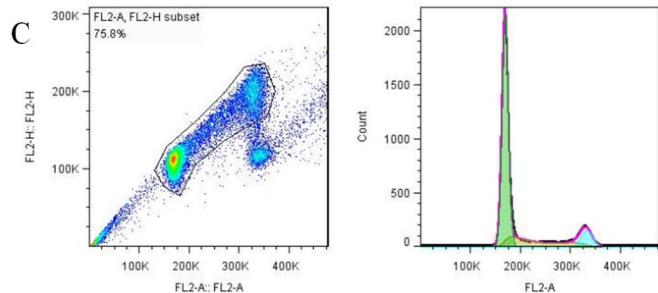

OV-RBFOX2

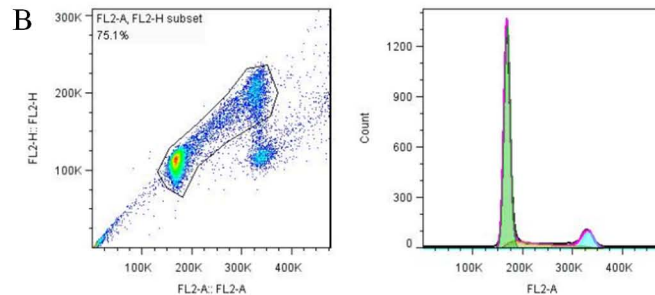

OV-MEF2D-V4

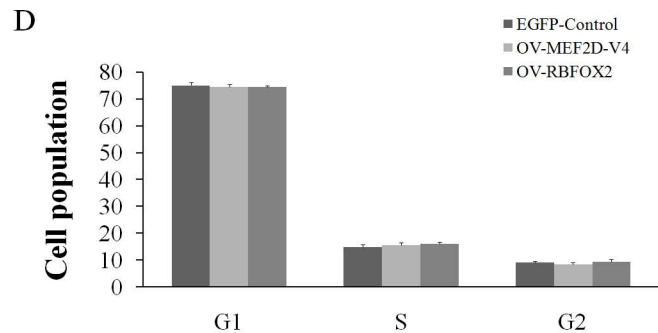

Supplement: Supplemental Information 5 — Flow Cytometry raw data of cell cycle analysis for myoblast transfected with EGFP-Control (A) or OV-RBFOX2 (B) or OV-MEF2D-V4 (C). (D) Statistical results of cell population. Bars represent S.E.M (n = 4). [file peerj-08-8351-s005.pdf]

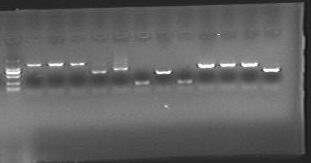

Supplement: Supplemental Information 7 [file peerj-08-8351-s007.zip › Uncropped blots/Uncropped blots of Fig 2A.png]

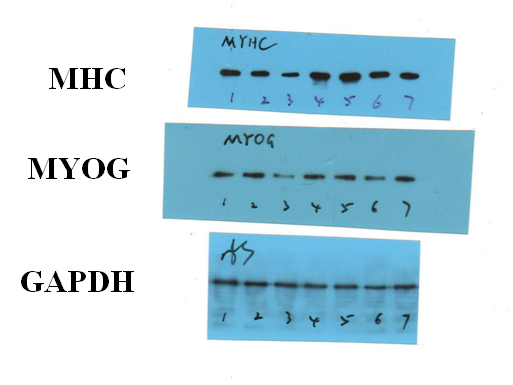

Supplement: Supplemental Information 7 [file peerj-08-8351-s007.zip › Uncropped blots/Uncropped blots of Fig 6B.png]
